# Identify QTLs and candidate genes underlying source-, sink-, and grain yield-related traits in rice by integrated analysis of bi-parental and natural populations

**DOI:** 10.1371/journal.pone.0237774

**Published:** 2020-08-14

**Authors:** Yun Wang, Junmin Wang, Laiyuan Zhai, Chengwei Liang, Kai Chen, Jianlong Xu

**Affiliations:** 1 Rice Research Institute, Shenyang Agricultural University, Shenyang, Liaoning, China; 2 The Institute of Crops and Nuclear Technology Utilization, Zhejiang Academy of Agricultural Sciences, Hangzhou, Zhejiang, China; 3 Institute of Crop Sciences/National Key Facility for Crop Gene Resources and Genetic Improvement, Chinese Academy of Agricultural Sciences, Beijing, China; 4 Shenzhen Branch, Guangdong Laboratory for Lingnan Modern Agriculture, Genome Analysis Laboratory of the Ministry of Agriculture, Agricultural Genomics Institute at Shenzhen, Chinese Academy of Agricultural Sciences, Shenzhen, Guangdong, China; Mahatma Phule Krishi Vidyapeeth College of Agriculture, INDIA

## Abstract

The source-sink relationship determines the ultimate grain yield of rice. In this study, we used a set of reciprocal introgression lines (ILs) derived from Xuishui09 × IR2061 to map quantitative trait loci (QTLs) that were associated with sink-, source-, and grain yield-related traits. A total of 95 QTLs influencing eight measured traits were identified using 6181 high-quality single nucleotide polymorphism markers. Nine background-independent QTLs were consistently detected in seven chromosomal regions in different genetic backgrounds. Seven QTLs clusters simultaneously affected sink-, source-, and grain yield-related traits, probably due to the genetic basis of significant correlations of grain yield with source and sink traits. We selected 15 candidate genes in the four QTLs consistently identified in the two populations by performing gene-based association and haplotype analyses using 2288 accessions from the 3K project. Among these, *LOC_Os03g48970* for *qTSN3b*, *LOC_Os06g04710* for *qFLL6a*, and *LOC_Os07g32510* for *qTGW7* were considered as the most likely candidate genes based on functional annotations. These results provide a basis for further study of candidate genes and for the development of high-yield rice varieties by balancing source–sink relationships using marker-assisted selection.

## Introduction

Rice (*Oryza sativa* L.) is one of the most important staple cereals in the world, especially in Asia. Due to continuously increasing population, diminishing cultivated area, and other factors, a nearly 40% increase in grain yield will be necessary to meet global food demand during the next 40 years [1]. Grain yield of rice is mainly determined by the coordination between sources and sinks [2]. Spikelet number per panicle and grain size are regarded as the major sinks that absorb and utilize photosynthate; tissues such as the topmost three leaves (especially the flag leaf) are often defined as the most important primary sources that produce photosynthate. A good balance between source strength and sink strength is a prerequisite for high yield potential.

To date, a number of efforts have been made to identify QTLs underlying source-, sink-, and yield-related traits by using genome-wide association and linkage analyses [3–5]. A large number of genes related to sources, sinks, and the source–sink relationship have been cloned, including sink-related genes, such as *Gn1a* [6], *DEP1* [7], *Ghd7* [8], *GNP6* [9], and *GNP1* [10] for spikelet number, *GW5* [11], *qTGW3* [12], and *GS3* [13] for grain size and grain weight, source-related genes such as *NAL9* [14] for leaf area, and source–sink relationship genes *NAL1* (*SPIKE*, *GPS*, and *LSCHL4*) [15–18]. Notably, several QTLs influencing source leaves (e.g., flag leaves) have been mapped to chromosomal regions closely affecting sink capacities (e.g., spikelet number per panicle and thousand-grain weight) and yield-related traits (e.g., panicle number), hinting at possible pleiotropy or tight linkage of genes/QTLs affecting source-, sink-, and yield-related traits [3, 4]. Detection of these QTLs is still crucial to gain a better understanding of the genetic basis and the relationship between source- and sink-related traits.

QTL mapping results largely depend on genetic background [19]. Many QTLs detected in mapping populations can’t be detectable in breeding population, thus impeding their application in molecular breeding. So identification of background-independent QTLs or integration of QTL mapping with molecular breeding in the same population will largely minimize effect of background on QTL detection. Genetic analysis using reciprocal introgression lines (ILs) derived from the same parents would be more helpful to understand complex traits [19–22], especially for studying genetic background effect on QTL detection [19–22]. In view of this consideration, we constructed a set of reciprocal introgression lines derived from Xuishui09 × IR2061 and identified background-independent QTLs underlying source-, sink-, and yield-related traits. We selected candidate genes from the important QTL intervals and validated the candidate genes based on gene-based association and haplotype analyses using 2288 rice accessions. Our study has shed new light on the genetic bases of source-sink related traits, and the results provide valuable information for molecular rice breeding by design for high yield potential.

## Materials and methods

### Materials

Xiushui09 is a high-yield semi-dwarf *japonica* (*geng*) rice variety from China while IR2061-520-6-9 (abbreviated as IR2061) is a drought tolerance *indica* (*xian*) breeding line introduced from the International Rice Research Institute (IRRI), Los Baños, Philippines. The two parents are genetically significant differences in grain yield, leaf trait, panicle trait, cold tolerance, drought tolerance and heat tolerance which are certainly worth of studying. Therefore, we chose Xuishui09 and IR2061 as the two parents to construct a set of reciprocal introgression lines (ILs) [21]. Specifically, the F_1_ plants were simultaneously backcrossed to the two parents to develop two BC_1_F_1_ populations, with each having around 100 plants. Twenty-five individuals randomly selected from BC_1_F_1_ plants were then used as the male parent to backcross with the corresponding parent to produce two BC_2_F_1_ populations. The BC progenies were selfed for five consecutive generations without selection, yielding lines from BC_2_F_2_ to BC_2_F_6_. Finally, two sets of ILs were developed, consisting of 226 BC_2_F_6_ lines in the Xiushui09 background (XS-ILs) and 233 BC_2_F_6_ lines in the IR2061 background (IR-ILs).

### Phenotypic investigation

All of the ILs and their parents were planted in Sanya (SY, 18.3°N, 109.3°E), Hainan province during December 2016–April 2017 and Hangzhou (HZ, 30.3°N, 120.2°E), Zhejiang province during May–October 2017. Each line was grown in a plot of two rows with 12 plants in each row at a spacing of 25 × 17 cm, with three replicates. The fields were managed according to the local standard practices. At the full heading stage, flag leaf width (FLW, in cm), flag leaf length (FLL, in cm) were measured on the main stems of five uniform plants from each replicate of each line. Flag leaf area (FLA, in cm^2^) was calculated as FLL × FLW × 0.75 [4]. At maturity, eight uniform plants in the middle row of each replicate of each line were sampled and dried in an oven at 70°C for five days followed by trait measurements of panicle number per plant (PN), total spikelet number per panicle (TSN), filled grain number per panicle (FGN), thousand-grain weight (TGW, in g), and grain yield per plant (GY, in g).

### Phenotypic data analysis

To dissect the effects of genotype-by-environment interaction (GEI) on the tested traits, variance components were estimated using multiple-site analysis, with all effects treated as random. Heritability across environments was computed using the estimated variance components as *V*_G_/(*V*_G_ + *V*_GEI_/*s* +*V*_e_/*sr*), where *V*_G_, *V*_GEI_, and *V*_e_ are the variances of genotype, genotype-by-environment interaction variance, and residual error, respectively; *s* is the number of environments, and *r* is the number of replicates. All of the analyses were conducted with the PBTools package (http://bbi.irri.org/products) developed by IRRI. A linear mixed model was used to integrate the phenotypic data from two environments; the line’s genotype effect was treated as the fixed effect, and the environment and replicate within the environment were treated as random effects. The lme4 in R package was used to calculate the best linear unbiased predictors (BLUPs) of each line. BLUP values of each line were used for analysis of phenotypic correlation using the *rcorr* function implemented in the R package Hmisc [23].

### Genotyping

Genomic DNA for SNP genotyping was isolated from approximately 100 mg fresh leaf samples of five-week-old seedlings from the two sets of ILs using a modified cetyltrimethylammonium bromide (CTAB) method [24]. Genotyping was performed using a customized rice 56 K SNP array containing 56,897 SNPs screened from the 3K Rice Genome Project [25, 26]. Target DNA preparation, chip hybridization, and array processing were conducted by Capital Bio-Technology (Beijing, China) according to the Affymetrix Axiom® 2.0 assay protocol. A three-step filtering strategy was applied to select high quality SNP markers from 56K SNP genotypic data for QTL mapping. Firstly, markers that were non-polymorphic between the two parents were removed. Secondly, all heterozygous genotypes were set as “missing” and markers with more than 10% missing values were removed. Thirdly, markers with a minor allele frequency (MAF) less than 3% were removed. Finally, 6181 high quality SNP markers were selected for QTL mapping for the two sets of ILs.

### QTL mapping

BLUP values of each line were used for QTL mapping of source-, sink-, and grain yield-related traits. A total of 6181 high-quality SNP markers were used to conduct the QTL identification. Linkage maps of XS-ILs and IR-ILs were constructed separately using the Map functionality in IciMapping [27]. Firstly, SNPs with a known chromosome ID on a physical map were anchored. The unanchored SNP markers were grouped based on a minimum logarithm of odds (LOD) score of 3. Secondly, a combined algorithm of nearest neighbor and two-opt algorithm of traveling salesman problem was used for marker ordering. Finally, the sum of adjacent recombination frequencies calculated with a five-marker window size was used as the rippling criterion. Recombination frequency was converted into map distance by the Kosambi mapping function. QTL mapping was performed using the software IciMapping V4.1 [27]. A probability level of the logarithm of the odds (LOD) value greater than 3.0 was used as the threshold for declaring the presence of a QTL.

### Identification of candidate genes

A total of 2288 worldwide accessions, which originated from 89 countries, generated from the 3,000 Rice Genomes Project [26] were collected to conduct gene-based association analysis and haplotype analysis to identify candidate genes. Based on the known population structure and division of subpopulations [26], the panel contained 1361 *xian* accessions (198 *xian*-1A, 177 *xian*-1B, 235 *xian*-2, 245 *xian*-3, and 506 *xian*-adm), 628 *geng* accessions (207 *geng* -tmp, 80 *geng*-sbtrp, 279 *geng*-trp, and 62 *geng*-adm), 176 *aus*/*boro*, 52 *basmati*/*sadri* and 71 highly admixture (*adm*) accessions (S1 Table). FLL, TGW, and TSN values of 2288 accessions were measured during December 2016–April 2017 in SY (S1 Table) using the same method as described above.

The following four steps were conducted to identify candidate genes for QTLs consistently detected in the two sets of ILs. Firstly, the genes located in the candidate regions for QTLs consistently detected in the two sets of ILs were extracted, and gene functions were annotated by the Rice Annotation Project Database (https://rapdb.dna.affrc.go.jp/). Then, all available SNPs located inside these genes were searched from 4.8 M SNPs data generated from 3K RGP in the Rice SNP-Seek Database [28]. The SNPs with a minor allele frequency less than 0.05 and/or a missing data rate exceeding 20% were removed, and the remaining high-quality SNPs located inside these genes were used to perform gene-based association analyses via compressed mixed linear model using principal component analysis (PCA) and kinship matrix (K). The analyses were performed with the Genome Association and Prediction Integrated Tool (GAPIT), a package of R software [29]. The critical *P*-value for declaring a significant marker-trait association was 10^−5^. Thirdly, haplotype analyses were conducted using all of the available non-synonymous SNPs located inside the gene CDS region searched from 18 M SNPs data generated from 3K RGP in the Rice SNP-Seek Database [28]. Candidate genes were determined by testing the significant differences among major haplotypes (containing more than 30 samples) for each important QTL through analysis of variance (ANOVA). Duncan’s multiple mean comparison test was carried out to determine the significance of any differences (5% significance level) using the agricolae package in R. Finally, the most likely candidate genes were selected based on their biologically related functions.

## Results

### Basic statistics of markers

The same set of 6181 well-distributed polymorphic SNP markers was available for genotyping of the two sets of ILs. The number of SNPs per chromosome ranged from 375 on chromosome 9 to 763 on chromosome 1 (S2 Table). The sizes of chromosomes varied from 23.0 Mb (chromosome 9) to 43.3 Mb (chromosome 1). The full genome size was 373.1 Mb, with an average marker spacing of 61.0 kb ranging from 55.7 kb on chromosome 2 to 72.8 kb on chromosome 12 (S2 Table).

### Genome compositions of the two sets of ILs

Most of the genomes of the reciprocal ILs were similar to their recurrent parents, with XS-ILs sharing on average 86.7% of the genome with the recurrent parent Xiushui09 (range from 44.6 to 100%), whereas IR-ILs shared on average 90.7% of the genome with the recurrent parent IR2061 (range from 58.5 to 100%) (S1 Fig). Obviously, the reciprocal introgression line populations were skewed toward one parent or the other, as expected.

### Phenotypic variation and correlation

**Fig 1:**
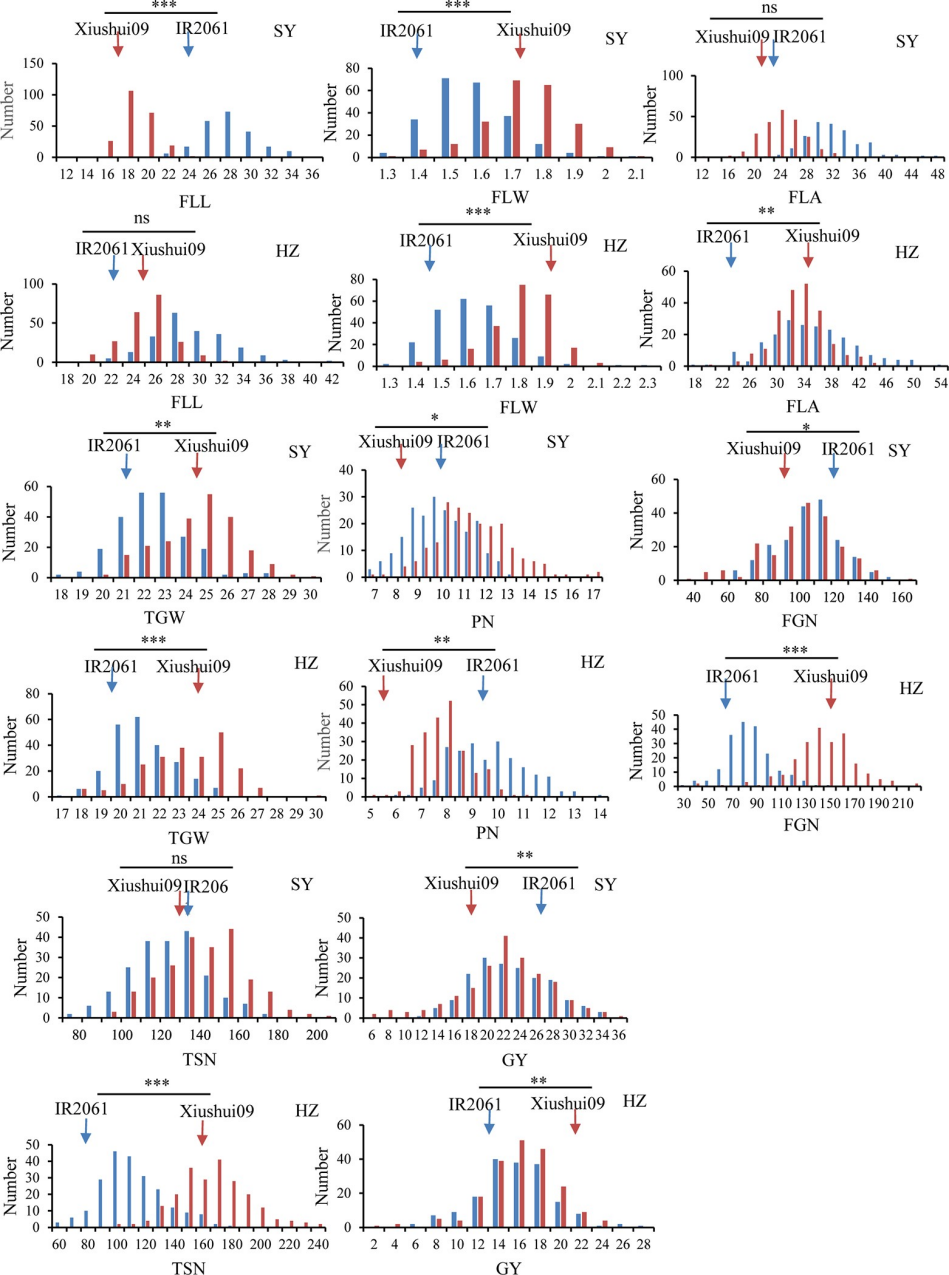
Frequency distribution of the eight evaluated traits in the reciprocal introgression lines across two environments. FLL, flag leaf length; FLW, flag leaf width; FLA, flag leaf area; PN, panicle number per plant; TSN, total spikelet number per panicle; FGN, filled grain number per panicle; TGW, thousand-grain weight; GY, grain yield per plant; SY, Sanya; HZ, Hangzhou; XS-ILs, introgression lines in Xiushui09 background; IR-ILs, introgression lines in IR2061 background; *, **, *** indicate significant differences between Xiushui09 and IR2061 at *P* < 0.05, *P* < 0.01, and *P* < 0.001, respectively; ns indicates non-significance.

Although Xiushui09 exhibited significantly greater FLW than IR2061, the FLA value did not differ between the two parents, because the former had significantly smaller FLL than the latter in SY (Fig 1). In HZ, Xiushui09 showed significantly larger FLA compared with IR2061, because the FLW of the former was significantly greater than that of the latter, and the FLL value did not differ between the two parents. The TGW of Xiushui09 was significantly higher than that of IR2061, whereas the PN of Xiushui09 was significantly lower than that of IR2061 in the two environments. In SY, Xiushui09 showed significantly lower FGN and similar TSN compared with IR2061, finally resulting in significantly lower GY compared with IR2061. In HZ, Xiushui09 exhibited significantly higher FGN and TSN, thus resulting in significantly higher GY compared with IR2061 (Fig 1). It was observed that the two sets of ILs had wide ranges of segregation, i.e., there were transgressive segregations for the eight traits (Fig 1).

In the two sets of ILs, five traits (FLL, FLW, FLA, TSN, and TGW) were controlled mainly by genotypes (*V*_G_), and the heritabilities of the five traits ranged from 0.38 for FLL to 0.70 for TGW, and from 0.33 for TSN to 0.64 for TGW in XS-ILs and IR-ILs, respectively. FGN, PN, and GY were more sensitive to environmental factors, with gene and environment together potentially affecting phenotypic variation. The heritability of the three traits ranged from 0.04 for GY to 0.23 for PN, and 0.14 for GY to 0.31 for FGN in XS-ILs and IR-ILs, respectively (S3 Table).

**Fig 2:**
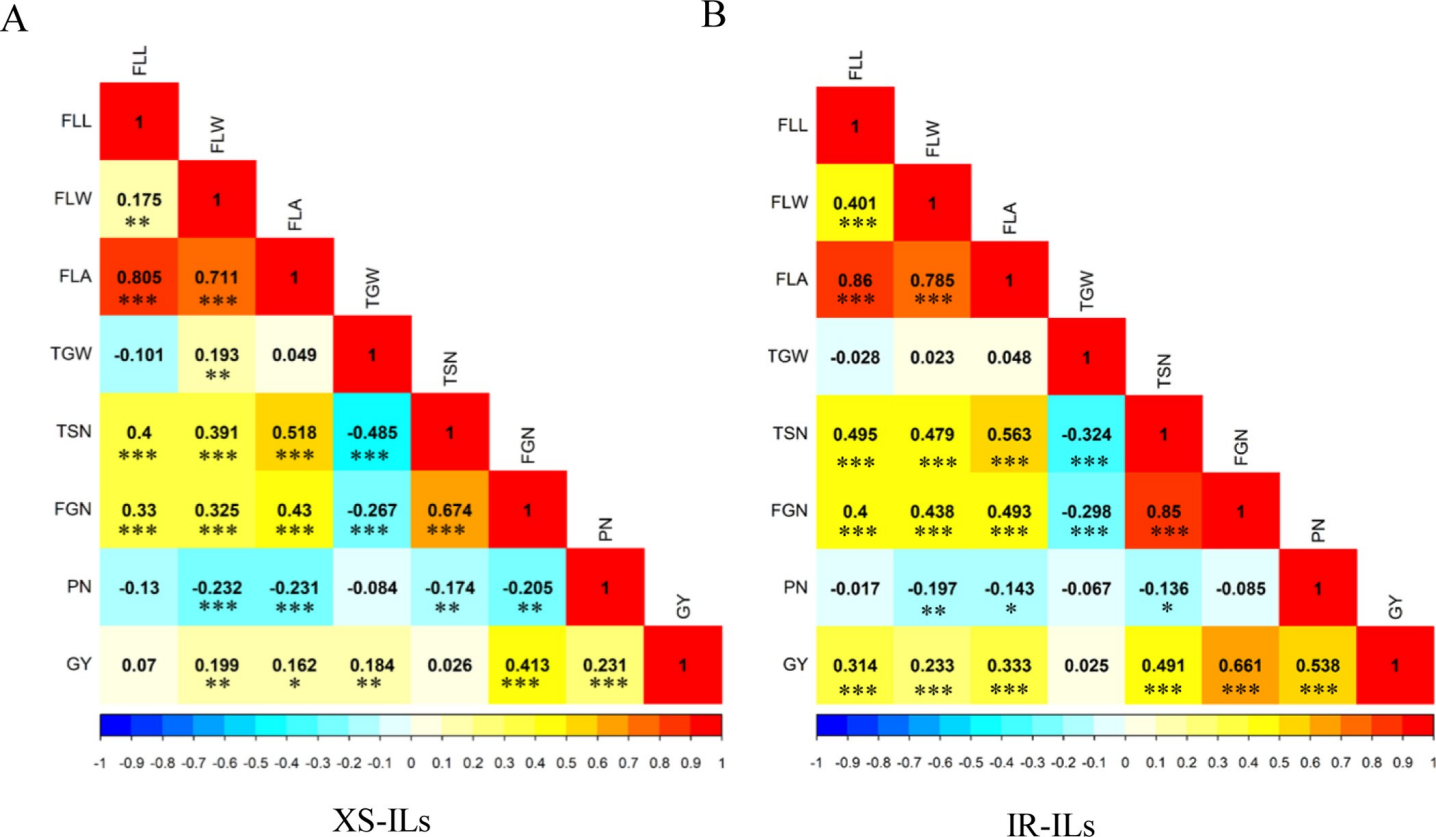
Correlations between the eight evaluated traits in introgression lines (ILs) in the Xuishui09 background (XS-ILs) (A) and the IR2061 background (IR-ILs) (B). FLL, flag leaf length; FLW, flag leaf width; FLA, flag leaf area; PN, panicle number per plant; FGN, filled grain number per panicle; TGW, thousand-grain weight; TSN, total spikelet number per panicle; GY, grain yield per plant. The colors of square correspond to values of the corresponding *r*. *, **, *** indicate significant correlations at *P* < 0.05, *P* < 0.01, and *P* < 0.001, respectively.

In the two sets of ILs, the trends of pairwise phenotypic correlations were similar (Fig 2). The FLA was significantly positively correlated with sink size (TSN) but strongly negatively correlated with PN, and there was no significant correlation with sink capacity (TGW). GY showed significant positive correlations with FGN and PN in the two populations. GY was also significantly positively correlated with TGW in XS-ILs, but there was no significant correlation with TGW in IR-ILs, implying that PN and FGN made larger contributions to GY than TGW in IR-ILs. There were negative or non-significant correlations among the three grain yield component traits (PN, FGN, and TGW) in the two populations. As expected, FLA showed significant positive correlations with its corresponding component traits (FLL and FLW), and it was also significantly positively correlated with GY in the two sets of ILs (Fig 2).

### QTL mapping

**Fig 3:**
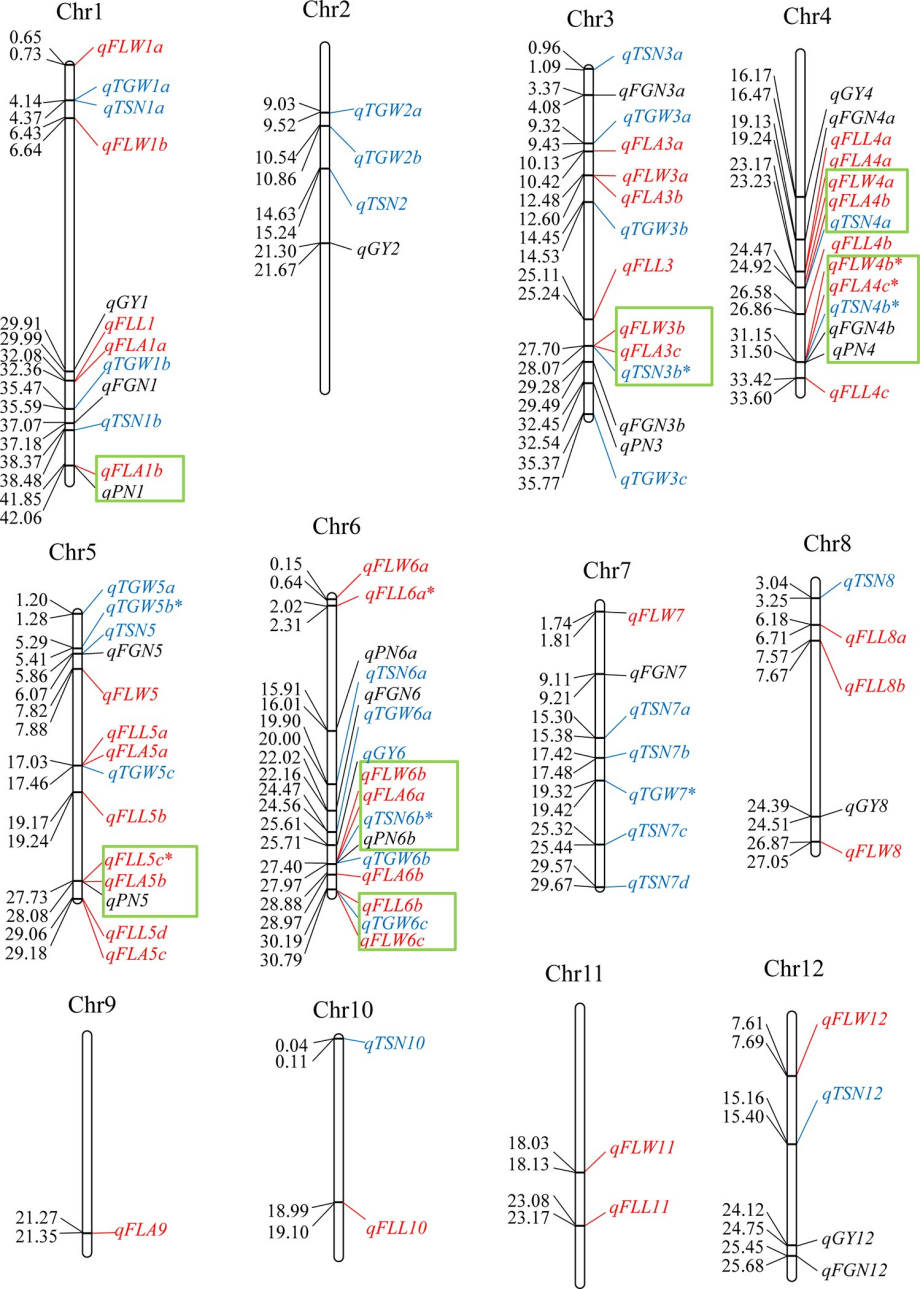
Distribution of QTLs identified in the two sets of introgression lines. Red, blue, and black indicate the QTLs affecting source-, sink-, and grain yield-related traits, respectively. Green rectangles indicate the QTL regions simultaneously affecting source-, and sink-/grain yield-related traits in the same population. Asterisks indicate that the QTLs were consistently identified in the two sets of introgression lines. Numbers on the chromosome indicate physical position of each QTL.

In total, 95 QTLs were identified for the eight yield-related traits in the two sets of ILs derived from the Xiushui09 × IR2061 cross, ranging from 6 for GY and PN to 17 for TSN. Among these, 45 QTLs were detected only in XS-ILs, and 41 only were found in IR-ILs. Nine QTLs in seven chromosomal regions were commonly identified in the two sets of ILs, with one QTL (*qFLW4b*) for FLW, two QTLs (*qFLL5c* and *qFLL6a*) for FLL, one QTL (*qFLA4c*) for FLA, two QTLs (*qTGW5b* and *qTGW7*) for TGW, and three QTLs (*qTSN3b*, *qTSN4b*, and *qTSN6b*) for TSN. The IR2061 alleles at the *qTGW7* and *qTSN6b* loci were associated with increased TGW and TSN, and they accounted for 4.6% and 4.3% of TGW, and 10.9% and 4.7% of TSN in XS-ILs and IR-ILs, respectively. The Xuishui09 alleles at the remaining loci resulted in increased corresponding trait values, and they accounted for 2.5% to 26.5% of the phenotypic variance (Table 1 and Fig 3).

**Table 1:** QTLs identified for yield-related traits in the reciprocal introgression lines.

Trait ^a^	QTL	Chr	Genetic interval ^c^ (cM)	Physical position (Mb)	XS-ILs ^b^			IR-ILs			Previously cloned gene
					LOD	A ^d^	*R*^*2*^(%)^e^	LOD	A	*R*^*2*^(%)	
FLW	*qFLW1a*	1	4.7–5.4	0.65–0.73	3.9	-0.04	2.5				
	*qFLW1b*	1	39.9–41.7	6.43–6.64				6.9	-0.05	6.9	
	*qFLW3a*	3	36.6–37.1	12.48–12.60	4.7	0.03	3.0				
	*qFLW3b*	3	111.0–116.4	27.70–28.07				3.9	0.03	4.0	
	*qFLW4a*	4	105.8–108.2	24.47–24.92				4.5	0.06	4.3	
	*qFLW4b*	4	146.0–148.6	31.15–31.50	3.9	-0.04	2.5	22.2	0.09	26.5	*NAL1* [17]
	*qFLW5*	5	60.2–62.1	7.82–7.88				3.3	-0.03	3.1	
	*qFLW6a*	6	1.7–3.9	0.15–0.64	6.1	0.03	3.9				
	*qFLW6b*	6	115.9–122.2	27.40–27.97	9.7	0.06	6.7				
	*qFLW6c*	6	137.4–139.8	30.19–30.79				3.2	-0.03	3.1	
	*qFLW7*	7	14.0–15.9	1.74–1.81	4.2	0.03	2.7				
	*qFLW8*	8	156.1–157.2	26.87–27.05	5.9	0.03	3.7				
	*qFLW11*	11	76.0–77.1	18.03–18.13				4.5	0.05	4.3	
	*qFLW12*	12	47.8–49.7	7.61–7.69				4.5	0.03	4.3	
FLL	*qFLL1*	1	189.6–191.1	32.08–32.36	5.9	0.8	6.6				
	*qFLL3*	3	76.1–78.0	25.11–25.24				7.0	1.9	8.4	
	*qFLL4a*	4	98.8–99.8	23.17–23.23	3.4	-0.5	3.6				
	*qFLL4b*	4	118.9–119.8	26.58–26.86				8.0	1.4	9.6	
	*qFLL4c*	4	160.8–164.2	33.42–33.60				3.2	0.9	3.7	
	*qFLL5a*	5	88.0–92.7	17.03–17.46				3.6	1.1	4.3	
	*qFLL5b*	5	104.4–104.8	19.17–19.24	10.0	1.0	11.8				
	*qFLL5c*	5	147.1–149.4	27.73–28.08	12.1	-1.3	14.2	11.6	1.8	14.5	
	*qFLL5d*	5	154.6–156.8	29.06–29.18	4.0	0.1	4.4				
	*qFLL6a*	6	10.9–12.0	2.02–2.31	9.6	-0.7	11.7	4.0	1.1	4.6	
	*qFLL6b*	6	137.4–139.8	30.19–30.79				5.7	-1.0	6.5	
	*qFLL8a*	8	61.7–63.4	6.18–6.71	12.4	-1.8	14.4				
	*qFLL8b*	8	65.6–66.2	7.57–7.67	22.6	2.2	29.5				
	*qFLL10*	10	52.8–53.2	18.99–19.10				5.3	-1.7	6.1	
	*qFLL11*	11	101.2–102.1	23.08–23.17	3.2	-0.4	3.5				
FLA	*qFLA1a*	1	189.6–191.1	32.08–32.36	4.1	1.2	4.2				
	*qFLA1b*	1	254.5–256.2	41.85–42.06				3.5	0.9	3.6	
	*qFLA3a*	3	27.8–28.0	10.13–10.42	3.0	1.0	3.1				
	*qFLA3b*	3	36.6–37.1	12.48–12.60	3.0	-0.3	3.4				
	*qFLA3c*	3	111.0–116.4	27.70–28.07				5.2	1.3	5.9	
	*qFLA4a*	4	98.8–99.8	23.17–23.23	3.2	-1.0	3.2				
	*qFLA4b*	4	105.8–108.2	24.47–24.92				3.3	2.4	3.7	
	*qFLA4c*	4	146.0–148.6	31.15–31.50	13.9	-2.0	15.7	11.3	2.7	13.9	*NAL1* [17]
	*qFLA5a*	5	88.0–92.7	17.03–17.46				4.4	1.8	4.9	
	*qFLA5b*	5	147.1–149.4	27.73–28.08	6.8	-1.9	7.1				
	*qFLA5c*	5	154.6–156.8	29.06–29.18				8.1	2.5	9.5	
	*qFLA6a*	6	115.9–122.2	27.40–27.97	5.2	1.6	5.9				
	*qFLA6b*	6	129.5–130.1	28.88–28.97				3.3	2.2	3.6	
	*qFLA9*	9	90.9–94.2	21.27–21.35				3.2	2.2	3.5	
TGW	*qTGW1a*	1	20.6–20.8	4.14–4.37	9.1	-1.5	8.5				
	*qTGW1b*	1	205.9–207.7	35.47–35.59				9.1	-0.7	7.4	
	*qTGW2a*	2	80.9–81.1	9.03–9.52	7.9	-0.8	7.3				
	*qTGW2b*	2	86.7–87.8	10.54–10.86				8.3	0.7	6.7	
	*qTGW3a*	3	24.6–26.1	9.32–9.43				6.4	1.2	5.2	
	*qTGW3b*	3	38.6–39.9	14.45–14.53				11.7	1.2	10.4	
	*qTGW3c*	3	196.9–198.4	35.37–35.77				3.5	0.4	2.9	*qTGW3* [12]
	*qTGW5a*	5	6.7–8.6	1.20–1.28				3.9	0.4	3.1	
	*qTGW5b*	5	36.7–38.6	5.29–5.41	22.5	-1.2	23.2	16.9	1.0	14.9	*GW5* [11]
	*qTGW5c*	5	88.0–92.7	17.03–17.46	3.7	0.4	3.1				
	*qTGW6a*	6	95.1–96.0	24.47–24.56	3.5	-0.7	3.1				
	*qTGW6b*	6	115.9–122.2	27.40–27.97				8.0	-0.8	6.7	
	*qTGW6c*	6	137.4–139.8	30.19–30.79				5.7	-0.7	4.5	
	*qTGW7*	7	108.9–109.6	19.32–19.42	5.1	0.8	4.6	5.4	-0.5	4.3	
TSN	*qTSN1a*	1	20.6–20.8	4.14–4.37	9.7	15.1	8.0				
	*qTSN1b*	1	224.0–225.3	38.37–38.48				4.1	5.4	4.2	
	*qTSN2*	2	102.9–104.6	14.63–15.24	5.0	8.2	4.0				
	*qTSN3a*	3	2.8–3.0	0.96–1.09	4.5	-8.7	3.5				
	*qTSN3b*	3	111.0–116.4	27.70–28.07	7.1	-10.3	5.7	5.5	5.2	5.8	
	*qTSN4a*	4	105.8–108.2	24.47–24.92				4.6	6.0	4.7	
	*qTSN4b*	4	146.0–148.6	31.15–31.50	9.7	-9.5	7.8	3.2	5.7	3.3	*NAL1* [17]
	*qTSN5*	5	41.0–42.7	5.86–6.07				3.6	-6.9	4.0	
	*qTSN6a*	6	70.9–72.4	19.9–20.00				6.4	-8.1	6.6	
	*qTSN6b*	6	115.9–122.2	27.40–27.97	12.9	15.3	10.9	4.4	-8.1	4.7	*APO1* [41]
	*qTSN7a*	7	78.4–79.5	15.30–15.38	6.9	-9.9	5.7				
	*qTSN7b*	7	97.5–98.8	17.42–17.48				7.7	7.7	8.2	
	*qTSN7c*	7	149.7–150.6	25.32–25.44				4.2	7.5	4.3	
	*qTSN7d*	7	177.0–178.7	29.57–29.67	3.1	-7.8	2.5				*Ghd7*.*1* [40]
	*qTSN8*	8	33.9–36.9	3.04–3.25	3.5	8.8	2.7				
	*qTSN10*	10	0–2.4	0.04–0.11				3.2	-10.8	3.3	
	*qTSN12*	12	109.8–112.2	15.16–15.40	3.5	-2.1	4.0				
FGN	*qFGN1*	1	213.0–213.5	37.07–37.18	4.8	-7.7	6.2				
	*qFGN3a*	3	10.1–12.1	3.37–4.08	7.4	-11.9	9.1				
	*qFGN3b*	3	145.9–146.9	29.28–29.49	4.3	-8.3	5.2				
	*qFGN4a*	4	63.7–64.4	19.13–19.24	9.9	-17.4	12.3				
	*qFGN4b*	4	146.0–148.6	31.15–31.50	5.5	-8.1	6.5				*NAL1* [17]
	*qFGN5*	5	41.0–42.7	5.86–6.07				5.3	-3.5	9.5	
	*qFGN6*	6	80.5–81.2	22.02–22.16				5.1	-8.5	8.7	
	*qFGN7*	7	61.5–62.1	9.11–9.21	3.2	-4.3	3.5				*Ghd7* [8]
	*qFGN12*	12	163.5–163.7	25.45–25.68	6.3	-7.8	7.3				
PN	*qPN1*	1	254.5–256.2	41.85–42.06				5.3	-0.5	9.6	
	*qPN3*	3	177.5–178.4	32.45–32.54	3.2	0.4	4.2				
	*qPN4*	4	146.0–148.6	31.15–31.50	7.5	0.6	10.8				*NAL1* [39]
	*qPN5*	5	147.1–149.4	27.73–28.08	5.0	0.6	7.0				
	*qPN6a*	6	52.6–54.1	15.91–16.01				4.1	0.5	7.3	
	*qPN6b*	6	115.9–122.2	27.40–27.97	3.1	-0.5	4.1				
GY	*qGY1*	1	181.7–183.2	29.91–29.99				4.3	0.5	8.9	
	*qGY2*	2	132.8–134.3	21.30–21.67	3.3	-1.1	4.7				
	*qGY4*	4	54.3–54.9	16.17–16.47	3.7	-1.9	5.6				
	*qGY6*	6	102.0–102.2	25.61–25.71				3.1	-1.8	5.9	
	*qGY8*	8	144.7–145.1	24.39–24.51	3.9	0.2	6.5				
	*qGY12*	12	156.2–160.3	24.12–24.75	8.7	-2.4	13.7				

^a^ FLL, flag leaf length; FLW, flag leaf width; FLA, flag leaf area; PN, panicle number per plant; TSN, total spikelet number per panicle; FGN, filled grain number per panicle; TGW, thousand-grain weight; GY, grain yield per plant.

^b^ XS-ILs, introgression lines in Xiushui09 background; IR-ILs, introgression line in IR2061 background.

^c^ Genetic interval indicate genetic distance in IR-ILs.

^d^ Additive effects (A) were estimated as the substitution effect of recurrent parent allele by donor parent allele.

^e^ *R*^2^ (%) is the proportion of phenotypic variance explained by each QTL.

### Chromosomal regions simultaneously harboring QTLs for source, sink, and grain yield traits

Comparisons of 43 QTLs affecting source flag leaf size (FLL, FLW, and FLA), 31 QTLs influencing sink capacities (TGW and TSN), and 21 QTLs affecting grain yield (GY) and its related traits (FGN and PN) revealed seven chromosomal regions simultaneously influencing source and sink/yield-related traits in the same population (Table 1 and Fig 3). These seven regions comprised the region of 41.85–42.06 Mb on chromosome 1 harboring *qFLA1b* for FLA and *qPN1* for PN detected in IR-ILs and the Xuishui09 alleles increased FLA at *qFLA1b* but decreased PN at *qPN1*; the region of 27.70–28.07 Mb on chromosome 3 harboring *qTSN3b*, *qFLW3b* and *qFLA3c* for TSN, FLW and FLA, respectively, detected in IR-ILs, and the Xuishui09 alleles at the three QTLs consistently resulted in increased corresponding trait values; the region of 24.47–24.92 Mb on chromosome 4 harboring *qFLW4a* for FLW, *qFLA4b* for FLA, and *qTSN4a* for TSN detected in IR-ILs, and the Xuishui09 alleles at the three QTLs consistently increased FLW, FLA, and TSN; the region of 31.15–31.50 Mb on chromosome 4 harboring *qFLW4b*, *qFLA4c*, *qTSN4b*, *qFGN4b*, and *qPN4* for FLW, FLA, TSN, FGN, and PN, respectively, detected in XS-ILs, and the Xuishui09 alleles resulted in decreased PN at *qPN4* but increased the corresponding phenotypic trait values at the remaining the four QTLs; the region of 27.73–28.08 Mb on chromosome 5 harboring *qFLL5c* for FLL, *qFLA5b* for FLA, and *qPN5* for PN detected in XS-ILs, and the Xuishui09 alleles increased FLL at *qFLL5c* and FLA at *qFLA5b* but decreased PN at *qPN5*; the region of 27.40–27.97 Mb on chromosome 6 harboring *qTSN6b*, *qFLW6b*, *qFLA6a*, and *qPN6b* for TSN, FLW, FLA, and PN, respectively, detected in XS-ILs, and the IR2061 alleles decreased PN at *qPN6b* but increased the trait values at the remaining three QTLs; the region of 30.19–30.79 Mb on chromosome 6 harboring *qFLL6b* for FLL, *qFLW6c* for FLW, and *qTGW6c* for TGW in IR-ILs, and the IR2061 alleles at three QTLs resulted in increased trait values (Fig 3).

### Performance of yield and its related traits of a near-isogenic IL XC10 and Xiushui09

**Fig 4:**
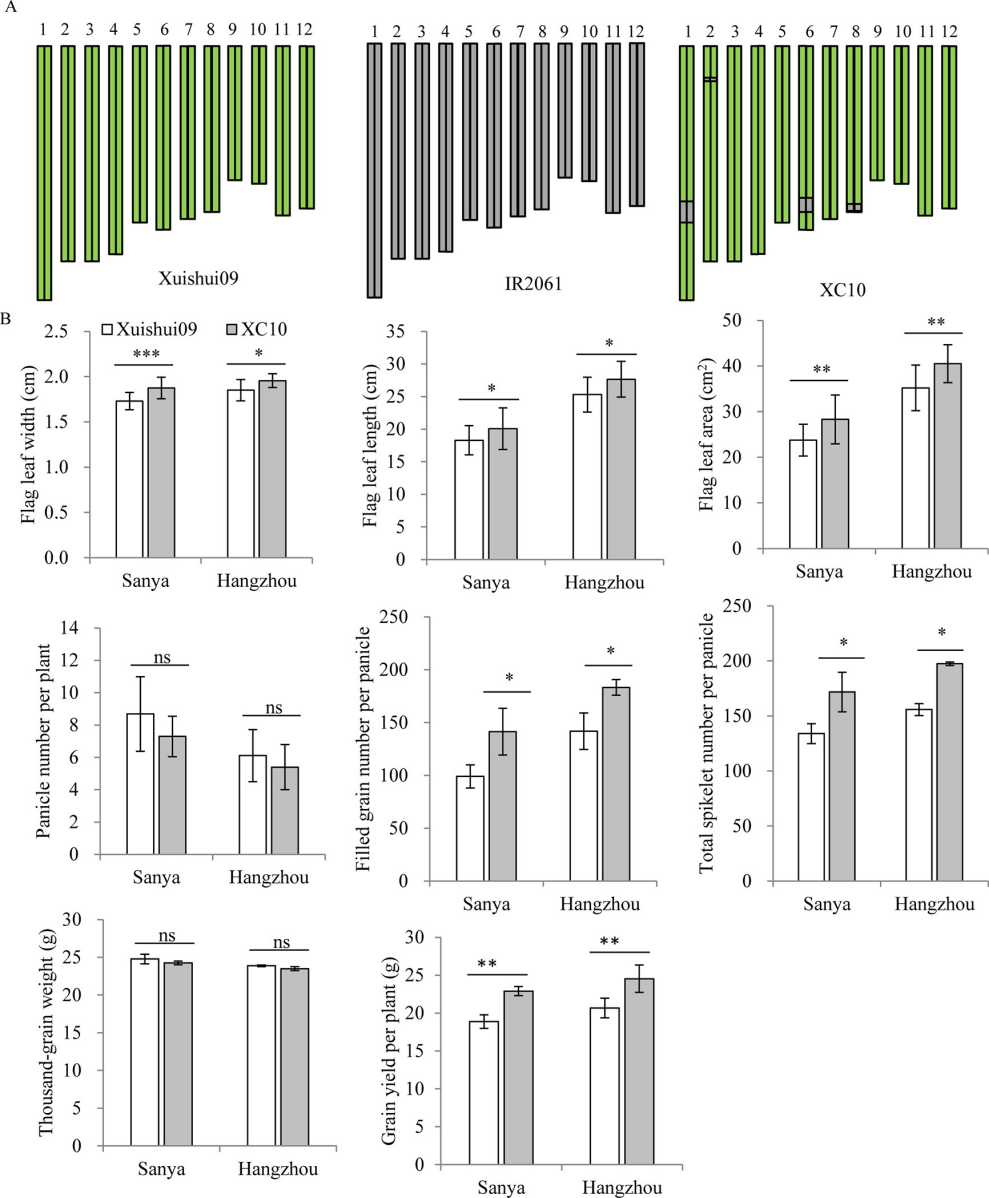
The performance of a near-isogenic introgression line XC10 and its recurrent parent Xuishui09. (A) Graphical genotype of Xuishui09, IR2061 and XC10, respectively. Yellow green and dark gray bars denote chromosome regions from Xuishui09 and IR2061, respectively. (B) Comparison of grain yield and its related traits between XC10 and Xuishui09 in Sanya and Hangzhou. *, **, *** indicate significant differences at *P* < 0.05, *P* < 0.01, and *P* < 0.001, respectively; ns indicates non-significance.

In this study, five chromosomal regions (27.70–28.07 Mb on chromosome 3, 24.47–24.92 Mb on chromosome 4, 31.15–31.50 Mb on chromosome 4, 27.40–27.97 Mb on chromosome 6, and 30.19–30.79 Mb on chromosome 6) simultaneously influencing source- and sink- related traits were identified in the same population (Fig 3). Among these chromosomal regions, the Xuishui09 alleles at the first three were associated with increased corresponding source-, and sink- trait values, whereas the IR2061 alleles at the latter two chromosomal regions increased trait values (Table 1). Based on selection against foreground favorable allele introgressions from the donor parent and background of the recurrent parent, one near-isogenic introgression line, XC10, was chosen from the XS-ILs by marker-assisted selection (MAS). Specifically, the IL XC10 carried the corresponding introgressed IR2061 alleles at *qTSN6b*/*qFLW6b*/*qFLA6a* located in the region of 27.40–27.97 Mb on chromosome 6 but had no IR2061 allele introgressions at the other QTLs for TSN, FLW, or FLA; otherwise the line showed 93.7% of the genome of Xuishui09 (Fig 4A). XC10 showed significant increases in FLA (+19.1% and 15.1%), FGN (+42.7% and 29.3%), and TSN (+28.2% and 26.7%) along with slightly lower but non-significant differences in TGW and PN compared with the recurrent parent Xuishui09 in SY and HZ. Finally, XC10 produced 21.3% and 18.7% higher grain yield than Xuishui09 in SY and HZ, respectively (Fig 4B), indicating that introgression of the IR2061 favorable alleles at *qTSN6b*/*qFLW6b*/*qFLA6a* contributed to increased grain yield due to increases in the corresponding source and sink traits.

### Candidate gene analysis

In this study, nine QTLs in the seven chromosomal regions were consistently identified in the two sets of ILs. We searched the Oryzabase online resource (https://shigen.nig.ac.jp/rice/oryzabase/) for known rice genes that co-localized with the nine QTLs. Three known genes (*APO1*, *NAL1*, and *GW5*) at five QTLs (*qTSN6b*, *qTSN4b*/*qFLA4c*/*qFLW4b*, and *qTGW5b*) were related to the corresponding traits.

**Fig 5:**
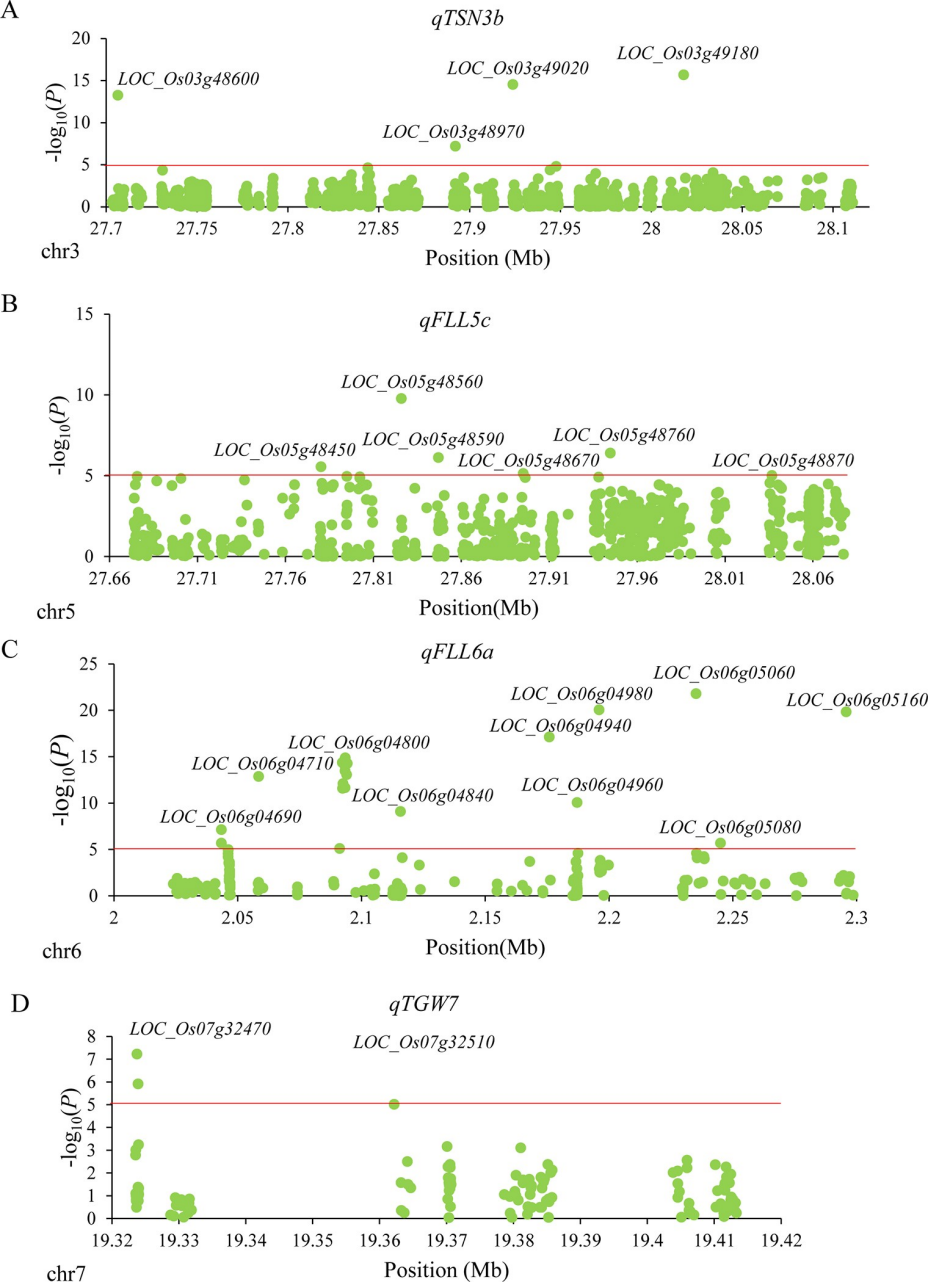
Gene-based association analyses of targeted genes related to *qTSN3b* (A), *qFLL5c* (B), *qFLL6a* (C) and *qTGW7* (D). Each green point show a SNP located in the genes in QTL intervals. Red lines show the threshold employed to determine significant SNPs.

We conducted gene-based association and haplotype analyses to identify candidate genes for the remaining four QTLs (*qFLL5c*, *qFLL6a*, *qTGW7*, and *qTSN3b*) using 2288 accessions of 3K. The 2288 accessions presented substantial variations for the FLL, TGW, and TSN in SY (S2 Fig). A total of 184 annotated genes located in the four QTL regions (*qFLL5c*, *qFLL6a*, *qTGW7*, and *qTSN3b*) according to the Rice Genome Annotation Project Database (RAP-DB). Of 2366 SNPs available in the 184 genes, a total of 32 significant SNPs located inside the 22 genes were identified to significantly associate with the corresponding traits (FLL, TSN, or TGW) by gene-based association analysis (Fig 5). Among the 22 genes, 15 genes had significant differences in corresponding traits among different haplotypes based on haplotype analyses (Table 2). Finally, the 15 genes were screened as candidate genes.

**Table 2:** Comparison of the yield-related traits among the haplotypes of the 22 genes within four QTLs consistently identified in the two sets of introgression lines.

QTL	MSU_locus	No. Hap	Hap1	Hap2	Hap3	Hap4	Hap5	Hap6	Hap7	Hap8	Hap9	MSU_locus_annotation
*qTSN3b*	*LOC_Os03g48600*	2	133.5b	144.8a	-	-	-	-	-	-	-	Domain of unknown function DUF966 domain containing protein, expressed
	*LOC_Os03g48970*	2	137.1a	130.1b	-	-	-	-	-	-	-	Nuclear transcription factor Y subunit, putative, expressed
	*LOC_Os03g49020*	5	135.1b	138b	130b	139.9b	158.9a	-	-	-	-	Hypothetical protein
	*LOC_Os03g49180*	3	136b	129.3c	145a	-	-	-	-	-	-	Alkaline phytoceramidase, putative, expressed
*qFLL5c*	*LOC_Os05g48450*	3	34.1a	34.7a	29.6b	-	-	-	-	-	-	Aminotransferase domain containing protein, putative, expressed
	*LOC_Os05g48560*	1	-	-	-	-	-	-	-	-	-	Expressed protein
	*LOC_Os05g48590*	6	34.9ab	33.5ab	27.6c	33.2b	35.2a	33.9ab	-	-	-	OsIAA19—Auxin-responsive Aux/IAA gene family member, expressed
	*LOC_Os05g48670*	2	33.3a	34a	-	-	-	-	-	-	-	OsFBT10—F-box and tubby domain containing protein, expressed
	*LOC_Os05g48760*	4	34.7a	34.8a	28.7c	32.8b	-	-	-	-	-	Protein of unknown function DUF1421 domain containing protein, expressed
	*LOC_Os05g48870*	4	33.5a	33.2b	28.1c	32.9b	-	-	-	-	-	Auxin response factor 15, putative, expressed
*qFLL6a*	*LOC_Os06g04690*	8	34.7a	30.7b	35a	32.5ab	34.4a	34.4a	33.3ab	33.1ab		OsFBX184—F-box domain containing protein, expressed
	*LOC_Os06g04710*	4	34.5a	31.8b	33.6a	25.6c	-	-	-	-	-	Expressed protein
	*LOC_Os06g04800*	3	33.7a	32.7a	32.3a	-	-	-	-	-	-	Peptidase, T1 family, putative, expressed
	*LOC_Os06g04840*	4	34.4a	32.7a	26.4b	33.1a	-	-	-	-	-	Leucine rich repeat protein, putative, expressed
	*LOC_Os06g04940*	1	-	-	-	-	-	-	-	-	-	Early nodulin 93 ENOD93 protein, putative, expressed
	*LOC_Os06g04960*	6	32a	34.4a	34.5a	32.6a	32.9a	33.6a	-	-	-	Expressed protein
	*LOC_Os06g04980*	5	34.4a	32a	32a	32.9a	32.9a	-	-	-	-	OsFBX185—F-box domain containing protein, expressed
	*LOC_Os06g05060*	4	34.6a	34.3a	28c	32.9b	-	-	-	-	-	ELF3 protein, putative, expressed
	*LOC_Os06g05080*	2	33.4a	33.3a	-	-	-	-	-	-	-	Cytochrome c oxidase subunit 5B, mitochondrial precursor, putative, expressed
	*LOC_Os06g05160*	9	34.7a	34ab	32.2abc	32.6abc	34.7a	31.4bc	33.2abc	31.6bc	30.7c	Sulfate transporter, putative, expressed
*qTGW7*	*LOC_Os07g32470*	8	23.9b	23.5b	26a	25.8a	23.5b	25.7a	25.9a	24.5b	-	Expressed protein
	*LOC_Os07g32510*	4	24.9a	24.9a	21.5b	22.2b		-	-	-	-	Dof zinc finger domain containing protein, putative, expressed

Letters (a, b, and c) are ranked by Duncan’s test at *P* < 0.05.

**Fig 6:**
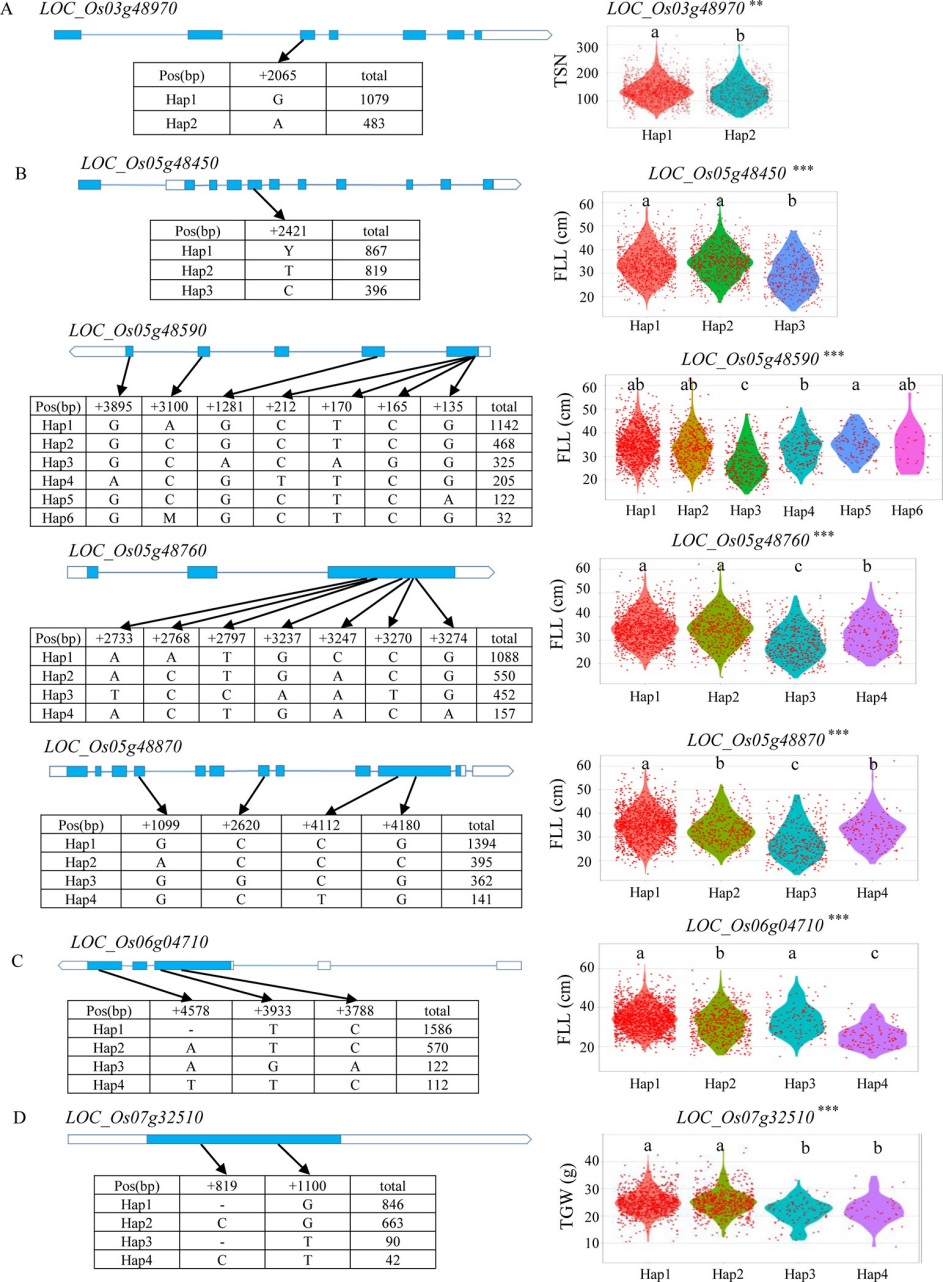
Haplotype analyses of the most likely three candidate genes for *qTSN3b*, *qFLL6a*, and *qTGW7*, and four candidate genes for *qFLL5c* for yield-related traits using non-synonymous single nucleotide polymorphisms (SNPs) within the coding region. (A) Gene structure (left) and total spikelet number per panicle of different haplotypes (right) of *LOC_Os03g48970* at *qTSN3b*. (B) Gene structures (left) and flag leaf length of different haplotypes (right) of *LOC_Os05g48450*, *LOC_Os05g48590*, *LOC_Os05g48760*, and *LOC_Os05g48870* at *qFLL5c*. (C) Gene structure (left) and flag leaf length of different haplotypes (right) of *LOC_Os06g04710* at *qFLL6a*. (D) Gene structure (left) and thousand-grain weight of different haplotypes (right) of *LOC_Os07g32510* at *qTGW7*. FLL, flag leaf length; TSN, total spikelet number per panicle; TGW, thousand-grain weight. The ** and *** denote significance of ANOVA at *P* < 0.01 and *P* < 0.001, respectively. Letters above violins (a, b and c) are ranked by Duncan’s test at *P* < 0.05.

For *qTSN3b*, this locus was mapped in the region of 27.70–28.07 Mb on chromosome 3 containing 63 annotated genes according to the Rice Genome Annotation Project Database (RAP-DB). A total of 1231 SNPs in 63 genes were used for association analysis. Based on gene-based association analysis results, four genes (*LOC_Os03g48600*, *LOC_Os03g48970*, *LOC_Os03g49020*, and *LOC_Os03g49180*) were used for haplotype analysis (Table 2). Significant differences in TSN were detected among different haplotypes for the four candidate genes (Table 2). Of these, *LOC_Os03g48970* is identical to *OsNF-YA4*, encoding a Nuclear Factor Y (NF-Y) transcription factor in rice [30]. Two haplotypes of *OsNF-YA4* were found, and Hap1 (G) was associated with significantly more TSN than Hap2 (A) (Fig 6A).

In the region from 27.73 to 28.08 Mb on chromosome 5 harboring *qFLL5c*, 750 SNPs in 60 genes were used for association analysis. According to the gene-based association analysis results, six genes (*LOC_Os05g48450*, *LOC_Os05g48560*, *LOC_Os05g48590*, *LOC_Os05g48670*, *LOC_Os05g48760* and *LOC_Os05g48870*) were selected for the haplotype analysis (Fig 5B). *LOC_Os05g48560* had only one haplotype containing more than 30 rice accessions. No significant differences in FLL were found among different haplotypes for *LOC_Os05g48670* (Table 2). Highly significant differences in FLL were detected among different haplotypes at the remaining four candidate genes (*LOC_Os05g48450*, *LOC_Os05g48590*, *LOC_Os05g48760*, and *LOC_Os05g48870*) (Table 2 and Fig 6B).

In the region from 2.02 to 2.31 Mb on chromosome 6 harboring *qFLL6a*, 249 SNPs in 51 genes were used for association analysis. Based on gene-based association analysis results, a total of 10 genes were used for haplotype analysis (Fig 5C). *LOC_Os06g04940* was identified in only one haplotype containing more than 30 rice accessions (Table 2). No significant haplotypes were found in *LOC_Os06g04800*, *LOC_Os06g04960*, *LOC_Os06g04980*, or *LOC_Os06g05080* (Table 2). Highly significant differences for FLL among different haplotypes were observed at five genes (*LOC_Os06g04690*, *LOC_Os06g04710*, *LOC_Os06g04840*, *LOC_Os06g05060* and *LOC_Os06g05160*) (Table 2). Among those genes, *LOC_Os06g04710* is identical to *dwarf and deformed flower 1* (*ddf1-1*), encoding an F-box protein in rice [31]. Four haplotypes of *ddf1-1* were found, and Hap 1 and Hap 3 exhibited significantly larger FLL than the other two haplotypes (Fig 6C).

The locus *qTGW7* was mapped in the region of 19.32–19.42 Mb on chromosome 7 containing 10 annotated genes. A total of 136 SNPs in 10 genes were used for association analysis. According to gene-based association analysis results, two genes (*LOC_Os07g32470* and *LOC_Os07g32510*) were used for haplotype analysis (Fig 5D). Significant differences in TGW were detected among different haplotypes at the two candidate genes (Table 2). *LOC_Os07g32510* encodes a Dof zinc finger domain protein. Four haplotypes of *LOC_Os07g32510* were found, and the TGW values of Hap 1 and Hap 2 were significantly larger than those of Hap 3 and Hap 4 (Fig 6D).

## Discussion

### Effect of genetic background on detection of grain yield QTLs

QTLs influencing grain yield and its related traits are usually sensitive to genetic background and environment [19]. Among the 95 QTLs affecting yield and its related traits identified in this study, only nine (9.5%) QTLs shared by the two different populations in the two different genetic backgrounds, indicating that most of the QTLs (90.5%) were very specific to the genetic background. The results clearly suggest that most yield QTLs detected in one population would not be identified in another population, i.e., highly genetic background effects existed that influenced the detection of QTLs for yield and related traits [19]. The QTL × genetic background interaction has been considered as one of the major factors limiting the use of QTLs in MAS for quantitatively inherited traits, as evidenced by effects of QTL *dep1* (*dense and erect panicle1*) on grain yield varied with the backgrounds [7, 32–34]. Therefore, it is indispensable to identify background-independent QTLs influencing yield for breeding high-yield rice by MAS, as shown in this study. Here, the nine QTLs (*qFLL5c*, *qFLL6a*, *qFLW4b*, *qFLA4c*, *qTSN3b*, *qTSN4b*, *qTSN6b*, *qTGW5b*, and *qTGW7*) consistently identified in the two different genetic backgrounds might certainly be made a priority for enhancement of rice production via MAS.

### Candidate gene identification of important QTLs

The region 27.70–28.07 Mb on chromosome 3 harboring *qTSN3b* contains four candidate genes, including *OsNF-YA4* (*LOC_Os03g48970*), encoding a Nuclear Factor Y (NF-Y) transcription factors in rice [30]. *OsNF-YA4* is involved in regulating the development of the rice panicle, affecting panicle number, grain number per panicle, grain filling, and grain weight. *OsNF-YA4* overexpressed transgenic plants exhibited abnormal yield component values, where grain filling rate, TSN, PN, and TGW were significantly decreased compared to the control [30]. In the present study, two haplotypes of *OsNF-YA4* were identified, and haplotype G showed significantly more TSN than haplotype A (Fig 6A). Therefore, *OsNF-YA4* (*LOC_Os03g48970*) is considered as the most likely candidate gene for *qTSN3b*.

For *qFLL6a*, *dwarf and deformed flower 1* (*ddf1-1*, *LOC_Os06g04710*) that encodes an F-box protein in rice is the most likely candidate gene. The *ddf1-1* mutant exhibited significantly shorter and thinner stems, leaves, and roots than the wild type [31]. Genetic complementation of the *ddf1-1* mutant showed that all the mutant phenotypes were rescued, including plants with dwarf stature and smaller stems, leaves, roots, and panicles, as well as aberrant spikelets [31]. In the present study, four haplotypes of *ddf1-1* were detected, of which Hap 1 and Hap 3 were associated with significantly larger FLL than the other two haplotypes (Hap 2 and Hap 4) (Fig 6C).

Of the two candidate genes for *qTGW7*, *LOC_Os07g32510* is a Dof zinc finger domain protein. The Dof proteins, a class of zinc finger domain transcription factors, have been reported to participate in regulation of gene expression in seed storage protein synthesis in developing endosperm [35, 36]. It has been reported that seed weight increased in *Arabidopisis* due to overexpressing one of two soybean *Dof* transcription factor genes *GmDof4* and *GmDof11* [37]. In rice, grain size and weight were decreased in the antisense transgenic lines of *Dof daily fluctuations 1* (*RDD1*) encoding a Dof transcription factor gene [38]. In the present study, four haplotypes of *LOC_Os07g32510* were detected, with Hap 1 and Hap 2 being associated with significantly larger TGW values than the other two haplotypes (Fig 6D), suggesting that *LOC_Os07g32510* is a likely candidate gene of *qTGW7* that probably affects TGW in rice.

### Comparison of QTLs detected in this study with previously cloned genes

Of the 95 QTLs for source-, sink- and grain yield-related traits, ten QTLs were located in the same or adjacent regions containing previously reported cloned genes in rice (Table 1). For example, *qFLW4b* for FLW, *qFLA4c* for FLA, *qTSN4b* for TSN, *qFGN4b* for FGN, and *qPN4* for PN were mapped in the region of 31.15–31.50 Mb on chromosome 4 that harbored the previously reported *NAL1* associated with pleiotropic effects on multiple traits related to the source (e.g., leaf width, leaf chlorophyll content, and photosynthetic efficiency), the sink (e.g., TSN), and the PN in diverse genetic backgrounds [15–18, 39]; *qTGW3c* in the region 35.37–35.77 Mb on chromosome 3 and *qTGW5b* in the region 5.29–5.41 Mb on chromosome 5 were co-located with *qTGW3* [12] and *GW5* [11] associated with rice grain size and grain weight, respectively; *qFGN7* in the region 9.11–9.21 Mb and *qTSN7d* in the region 29.57–29.67 Mb on chromosome 7 were close to *Ghd7* [8] and *Ghd7*.*1* [40] regulating spikelet number, plant height and heading date in rice; *qTSN6b* for TSN was co-located in the region 27.40–27.97 Mb on chromosome 6 harboring *APO1* for the number of primary rachis branches that ultimately increase the harvest index and grain yield in rice [41]. Allelism of the above QTLs for yield and its related traits detected in this study with the previously reported genes awaits further verification using fine mapping and QTL cloning.

### Application in rice breeding for high yield potential

In the history of rice breeding, the balance between sources (e.g., FLL, FLW, and FLA) and sinks (e.g., TSN and TGW) plays an important role in guiding the breeding of super high-yield rice varieties. The significant positive correlations of FLL, FLW and FLA with TSN in the two populations were in agreement with previous studies [4]. However, no significant correlations were observed between the three flag leaf traits and TGW except between FLW and TGW in XS-ILs in the two sets of ILs (Fig 2). In this study, we identified five QTL clusters (in the regions of 27.70–28.07 Mb on chromosome 3, 24.47–24.92 Mb on chromosome 4, 31.15–31.50 Mb on chromosome 4, 27.40–27.97 Mb on chromosome 6, and 30.19–30.79 Mb on chromosome 6) simultaneously influencing the source- and sink-related traits, hinting at possible pleiotropy or tight linkage. *SPIKE* (*NAL1*) located at a QTL cluster (*qFLW4b*/*qFLA4c*/*qTSN4b*/*qFGN4b*) in the region of 31.15–31.50 Mb on chromosome 4 showed pleiotropic effects in regulating the development of multiple traits related to source (leaf width, leaf chlorophyll content, and photosynthetic efficiency) and sink (spikelet number per panicle) in different genetic backgrounds [15–18]. NILs with the superior *NAL1* allele achieved 18.7% and 18% yield increases compared with their controls 9311 and IRRI146 [15, 17], respectively. Therefore, we inferred that it is possible to improve the grain yield by introgression of favorable alleles of the above five QTL clusters (*qFLW3b*/*qFLA3c*/*qTSN3b*, *qFLW4a*/*qFLA4b*/*qTSN4a*, *qFLW4b*/*qFLA4c*/*qTSN4b*/*qFGN4b*, *qFLW6b*/*qFLA6a*/*qTSN6b*, and *qFLW6c*/*qFLL6b*/*qTGW6c*) into *geng* or *xian* varieties to develop super high-yield rice cultivars, as demonstrated by the IL XC10 from this study (Fig 4).

In general, large-panicle cultivars exhibit greater plant size, larger FLL and FLW, and fewer numbers of tillers [42]. In the present study, strong negative correlations of FLL, FLW, FLA and TSN with PN were observed in the two populations (Fig 2). The significant negative correlation between TSN and PN, which is also known as the concept ‘yield component compensation’ can be partly explained by the developmental trade-off between tiller or panicle number and organ size (plant height, leaf size, and panicle size) or physiological competition for nitrogen during spikelet differentiation in rice [43, 44]. In this study, we identified two QTL clusters in the region of 31.15–31.50 Mb on chromosome 4 and 27.40–27.97 Mb on chromosome 6 underlying the development of multiple traits related to source (FLW and FLA), sink (TSN), and grain yield-related traits (PN and FGN) in XS-ILs, and the alleles increasing FLW, FLA, TSN, and FGN decreased PN. In addition, another two QTL clusters in the region of 41.85–42.06 Mb on chromosome 1 and 27.73–28.08 Mb on chromosome 5 were identified that simultaneously influenced leaf size and PN with opposite directions of additive effect. However, only a few overlapping QTL clusters affecting PN and the other source- and sink-related traits were detected in the two populations, suggesting that most QTLs influencing PN and the other source- and sink-related traits are genetically independent. Therefore, one strategy for improvement of yield could be via deploying or pyramiding different favorable alleles of independent QTLs for PN and the other source- and sink-related traits to well coordinate source and sink relationships.

## Conclusions

We identified 95 QTLs for the yield- traits, nine of which were consistently detected in seven chromosomal regions in the two set of ILs, and seven QTL clusters that simultaneously affected source-, sink-, and grain yield-related traits were detected in the same population. A total 15 candidate genes for the four QTLs consistently detected in the two populations were identified and the most likely candidate genes for three QTLs (*qTSN3b*, *qFLL6a*, and *qTGW7*) were inferred from gene-based association analysis, haplotype analysis and functional annotation. Favorable alleles of genetic background-independent QTLs for source-, sink, and grain yield-related traits were mined. These results provide an effective guide to developing varieties with high yield potential by balancing source–sink dynamics via marker-assisted selection.

## Supporting information

S1 Table Details of the 2288 accessions investigated and raw data of yield related traits measured in Sanya. (XLSX)

S2 Table Distribution of SNP markers on 12 chromosomes of rice. (PDF)

S3 Table Variance components and heritabilty estimated by multiple-site analysis. (PDF)

S1 Fig Frequency distribution of the Xuishui09 genome in the reciprocal Introgression Lines (ILs) in Xuishui09 (XS) and IR2061 (IR) backgrounds. (PDF)

S2 Fig Box plots of flag leaf length, thousand-grain weight, and total spikelet number per panicle in 2288 accessions. (PDF)
