# Supplementary material for: Identify QTLs and candidate genes underlying source-, sink-, and grain yield-related traits in rice by integrated analysis of bi-parental and natural populations
Source: PLoS One. 2020 Aug 14;15(8):e0237774. doi: 10.1371/journal.pone.0237774 (PMC7428182; doi:10.1371/journal.pone.0237774)
Supplement: S2 Table — (PDF) [file pone.0237774.s002.pdf]

S2 Table. Distribution of SNP markers on 12 chromosomes of rice.

Chromosome	No. of markers	Size (Mb)	Average distance (kb)
1	763	43.3	56.7
2	644	35.9	55.7
3	636	36.4	57.2
4	565	35.5	62.8
5	501	30.0	59.9
6	482	31.2	64.7
7	488	29.7	60.9
8	482	28.4	58.9
9	375	23.0	61.3
10	412	23.2	56.3
11	455	29.0	63.7
12	378	27.5	72.8
Total/average	6181	373.1	61.0
