# Supplementary material for: Identify QTLs and candidate genes underlying source-, sink-, and grain yield-related traits in rice by integrated analysis of bi-parental and natural populations
Source: PLoS One. 2020 Aug 14;15(8):e0237774. doi: 10.1371/journal.pone.0237774 (PMC7428182; doi:10.1371/journal.pone.0237774)
Supplement: S3 Table — (PDF) [file pone.0237774.s003.pdf]

S3 Table. Variance components and heritabilty estimated by multiple-site analysis

Trait	XS-ILs						IR-ILs					
	V_{GEI}	V_G	Rep (Env)	V_E	V_e	h^2	V_{GEI}	V_G	Rep (Env)	V_E	V_e	h^2
FLL	1.28	1.75	0.01	19.70	1.73	0.38	2.54	5.66	0.03	1.14	4.25	0.50
FLW	0.00	0.01	0.00	0.00	0.00	0.59	0.01	0.01	0.00	0.00	0.01	0.43
FLA	3.60	6.66	0.60	40.26	4.69	0.48	10.59	17.31	0.26	4.99	8.36	0.49
PN	0.77	0.60	0.02	7.51	1.10	0.23	0.81	0.64	0.06	0.18	1.28	0.22
TSN	149.62	309.56	1.99	170.66	182.61	0.52	175.25	198.34	6.60	170.26	121.21	0.33
FGN	346.44	168.54	7.37	638.46	166.26	0.19	166.36	137.42	2.78	366.10	103.20	0.31
TGW	0.66	2.89	0.00	0.79	0.91	0.70	0.50	1.80	0.09	0.78	0.77	0.64
GY	12.70	2.13	0.86	18.69	11.24	0.04	6.48	2.94	0.08	74.01	6.20	0.14

FLL, flag leaf length; FLW, flag leaf width; FLA, flag leaf area; PN, panicle number per plant; TSN, total spikelets number per panicle; FGN, filled grain number per panicle; TGW, thousand-grain weight; GY, grain yield per plant; XS-ILs, introgression lines in Xiushui09 background; IR-ILs, introgression line in IR2061 background; V_{G}, genotypic variance; V_{GEI}, genotype-by-environment interaction variance; V_{e}, residual variance; Rep (Env), replication variance within environment; h^2, narrow-sense heritability.
