# Supplementary material for: Identify QTLs and candidate genes underlying source-, sink-, and grain yield-related traits in rice by integrated analysis of bi-parental and natural populations
Source: PLoS One. 2020 Aug 14;15(8):e0237774. doi: 10.1371/journal.pone.0237774 (PMC7428182; doi:10.1371/journal.pone.0237774)
Supplement: S1 Fig — (PDF) [file pone.0237774.s004.pdf]

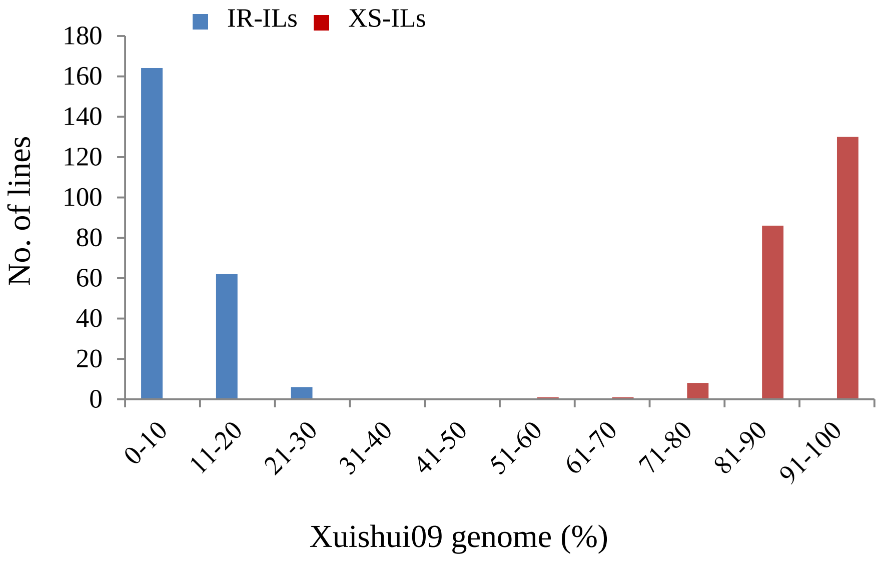

S1 Fig. Frequency distribution of the Xuishui09 genome in the reciprocal introgression lines (ILs) in Xuishui09 (XS) and IR2041 (IR) backgrounds.
