# Supplementary material for: Identify QTLs and candidate genes underlying source-, sink-, and grain yield-related traits in rice by integrated analysis of bi-parental and natural populations
Source: PLoS One. 2020 Aug 14;15(8):e0237774. doi: 10.1371/journal.pone.0237774 (PMC7428182; doi:10.1371/journal.pone.0237774)
Supplement: S2 Fig — (PDF) [file pone.0237774.s005.pdf]

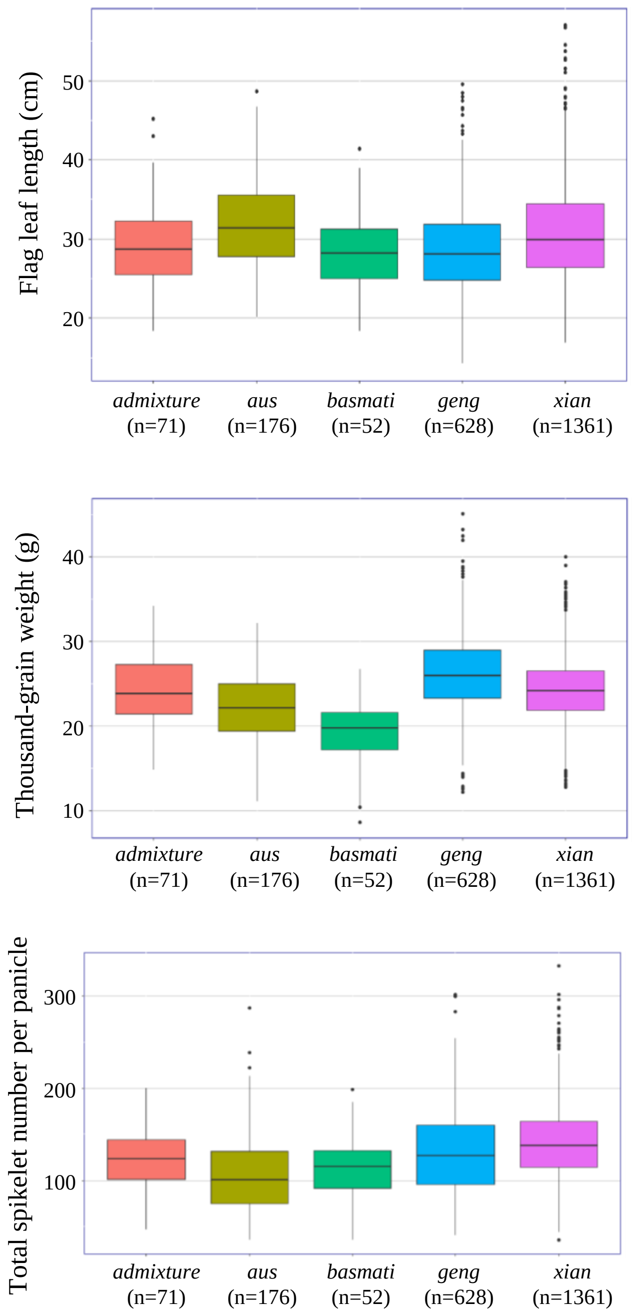

S2 Fig. Box plots of flag leaf length, thousand-grain weight, and total spikelet number per panicle in 2288 accessions.
